# Supplementary material for: Coherent charge oscillations in a bilayer graphene double quantum dot
Source: Nat Commun. 2023 Nov 30;14:7911. doi: 10.1038/s41467-023-43541-3 (PMC10689829; doi:10.1038/s41467-023-43541-3)
Supplement: Supplementary file 1 — Supplementary Information [file 41467_2023_43541_MOESM1_ESM.pdf]

Supplementary Information
Coherent Charge Oscillations
in a Bilayer Graphene Double Quantum Dot

K. Hecker*,[1,2] L. Banszerus*,[1,2] A. Schäpers,[1] S. Möller,[1,2] A. Peters,[1]
E. Icking,[1,2] K. Watanabe,[3] T. Taniguchi,[4] C. Volk,[1,2] and C. Stampfer[1,2]

[1]*JARA-FIT and 2nd Institute of Physics,*
RWTH Aachen University, 52074 Aachen, Germany, EU

[2]*Peter Grünberg Institute (PGI-9), Forschungszentrum Jülich, 52425 Jülich, Germany, EU*

[3]*Research Center for Functional Materials,*
National Institute for Materials Science,
1-1 Namiki, Tsukuba 305-0044, Japan

[4]*International Center for Materials Nanoarchitectonics,*
National Institute for Materials Science,
1-1 Namiki, Tsukuba 305-0044, Japan

(Dated: November 1, 2023)

I. GATE LEVER ARM

The lever arm α converting the voltage applied to the left FG, V_L, into the detuning energy ε can be determined by fitting Eq. (1) in the main manuscript to the photon assisted tunneling (PAT) data (see Fig. 2h of the main manuscript). Supplementary Fig. 1 shows a finite bias charge stability diagram of a triple point where the dashed black lines mark a gate voltage range V_{SD}/α. The outline of the triple point matches very well the independently evaluated lever arm from the PAT measurements.

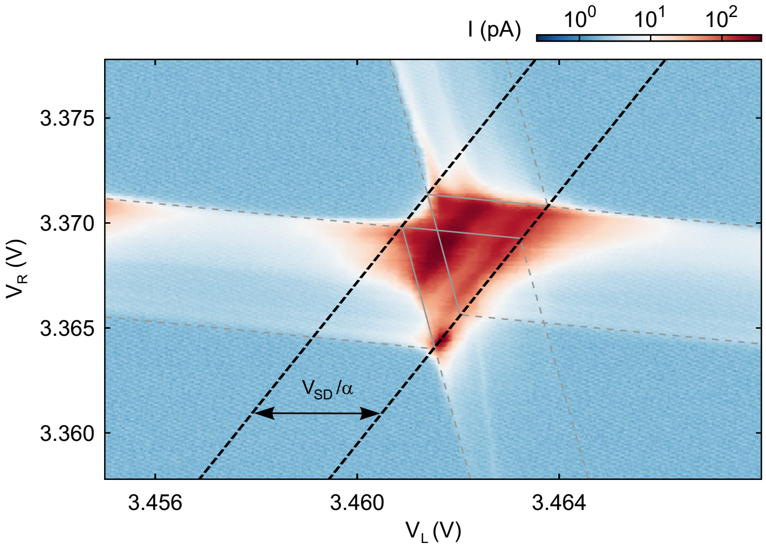

Supplementary Fig. 1. **Charge stability diagram and lever arm.** Charge stability diagram as shown in Fig. 2a in the main manuscript with a finite bias voltage of $V_{SD} = 0.5$ mV applied. The black dashed lines indicate the extension of the bias window in detuning, using the lever arm α extracted from the PAT data shown in Fig. 2h in the manuscript. The gray lines are guides to the eye, highlighting the outline of the tripe point pair and regions of co-tunneling.

II. COMPLEMENTARY DATA ON PHOTON-ASSISTED TUNNELING

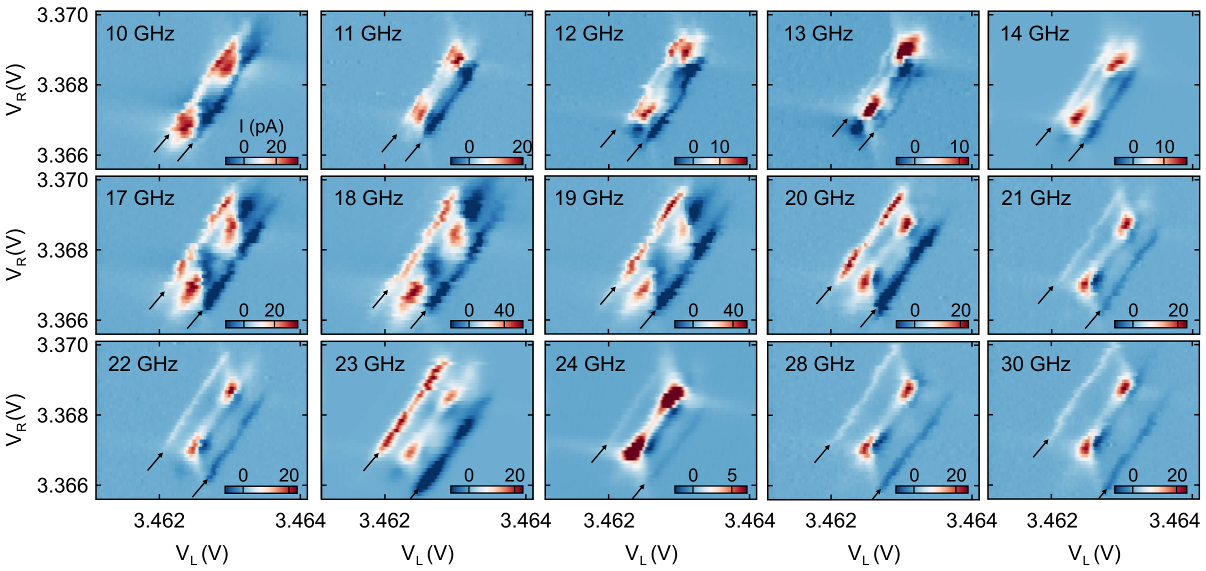

Supplementary Fig. 2. **Photon assisted tunneling for varying frequencies.** Complementary data of Fig. 2d-f in the main manuscript measured in a frequency range of $f = 10 - 30\,$GHz. Black arrows are highlighting the positions of the PAT resonances shifting on the detuning axis with the applied frequency, f.

Photon-assisted tunneling (PAT) spectroscopy can be used to determine the interdot tunnel coupling $\Delta/(2h)$ and the ensemble charge decoherence time $T^*_{2,\mathrm{PAT}}$. For that purpose, the power of the microwave excitation has been optimized to achieve a sufficient signal-to-noise ratio while avoiding multi-photon absorption processes [1]. Supplementary Fig. 2 shows a set of charge stability diagrams of a triple point with an applied microwave excitation ranging from $f = 10\,$GHz to $f = 30\,$GHz (c.f. Figs. 2d-f in the main manuscript). The black arrows highlight the splitting of the PAT peaks increasing with the applied microwave excitation frequency.

Higher order PAT resonances that emerge as the microwave power is increased are shown in Supplementary Fig. 3a. Here, a line cut as function of finger gate voltage, V_L, is shown as a function of applied power. The elevated power level renormalizes the tunnel coupling by the squared Bessel function and can be described by the function $f = \sqrt{(\alpha\delta V_\mathrm{L})^2 + J_0(a)^2\Delta^2}/h$, where $a = e\beta V_\mathrm{rms}/hf$. In the case of low applied power ($a \ll 1$), $J_0^2 \approx 1$, the tunnel coupling

can be estimated directly by fitting Eq. 1 in the main manuscript [?]. Supplementary Fig. 3b displays the averaged current across the PAT resonances of orders $n = 1, 2, 3$. A comparison with the inset, presenting the squared Bessel functions, reveals the anticipated proportionality of higher order PAT [?]. The power for the evaluation of Δ is set to a minimum, where only the first order PAT process is visible.

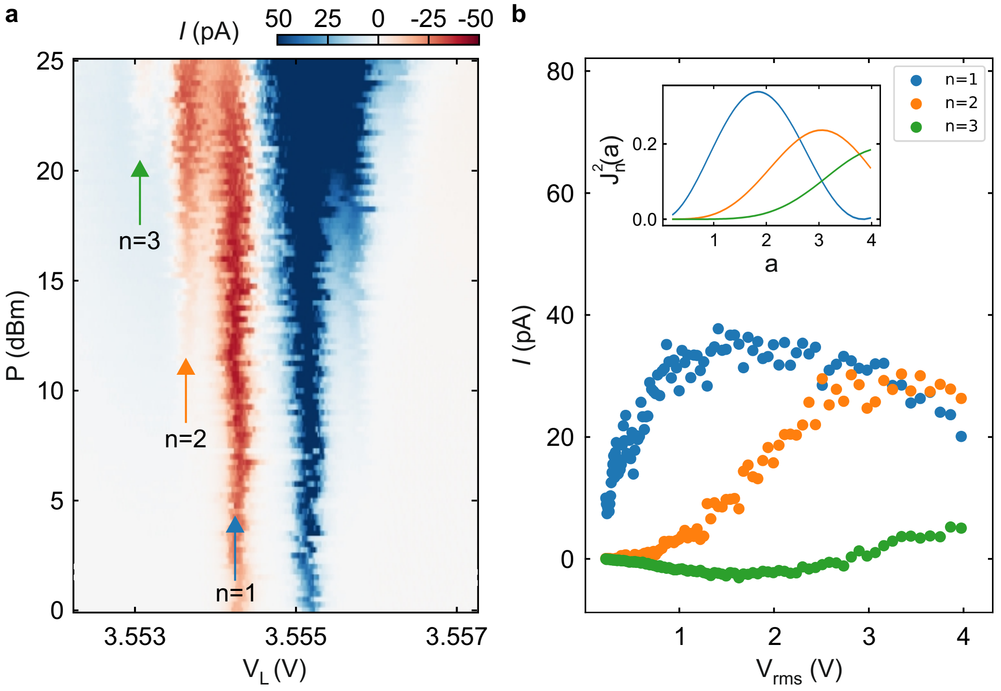

Supplementary Fig. 3. **Line trace as function of the applied microwave power a** Line cut through a triple point at $V_{\mathrm{R}} = 3.3601\,\mathrm{V}$, $V_{\mathrm{SD}} = 5\,\mu\mathrm{V}$ as function of the power of the applied sine pulse with a frequency of $f = 23\,\mathrm{GHz}$. **b** Averaged current through the DQD system along the PAT peaks of the first, second and third order (n=1,2,3) as function of the effective power V_{rms}. Inset: Squared Bessel function of the first to third order as function of the parameter $a = e\beta V_{\mathrm{rms}}/hf$.

Supplementary Fig. 4 shows line cuts through the triple point measured at $f = 20\,\mathrm{GHz}$. Lorentzian line shapes are fitted to the positive (red dashed line) and negative (blue dashed line) PAT peaks (c.f. Fig. 2g in the main manuscript). The shown fits are used to evaluate the peak separation at different V_{R}. For each frequency, f, $2\delta V_{\mathrm{L}}$ is found by averaging the peak separation of several line traces. The tunnel coupling $\Delta/(2h)$ is determined as described in the main manuscript (see Eq. (1) and Fig. 2h). An estimate of the ensemble charge decoherence time can be extacted from the FWHM, γ, of the Lorentzian peaks according to $T^*_{2,\mathrm{PAT}} = 2h/(\alpha\gamma)$ [2, 3]. The decoherence times evaluated from several line traces of the

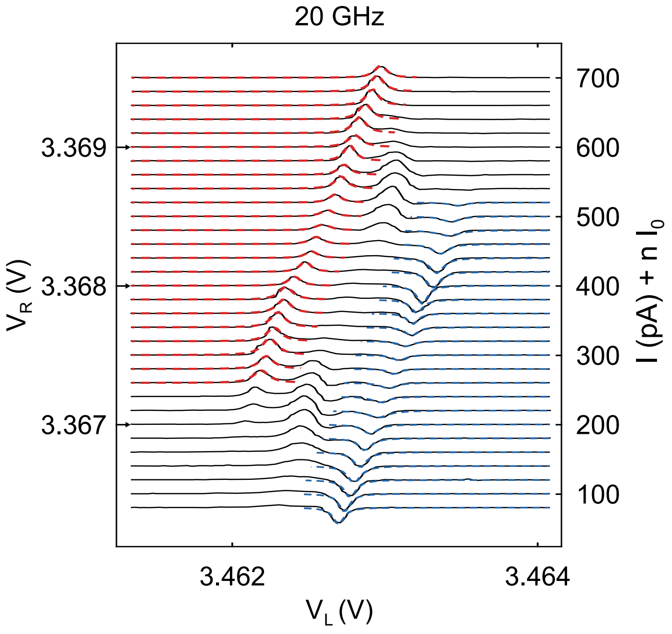

Supplementary Fig. 4. **Line traces with fits.** Line traces of the data shown in Supplementary Fig. 2 at a frequency of $f = 20\,\mathrm{GHz}$. The dashed lines show fits to the negative (blue) and positive (red) PAT peaks. The left y-axis marks position of the line trace on the V_R-axis, while the right y-axis shows the current, $I + nI_0$, with $I_0 = 20\,\mathrm{pA}$ and the index of the trace, n.

data in Supplementary Fig. 2 and Fig. 2d-f are shown as a histogram in Fig. 5f in the main manuscript.

III. COMPLEMENTARY DATA ON COHERENT OSCILLATIONS

This section presents further data on the Landau-Zener-Stückelberg (LZSM) interference pattern to elucidate the effect of different measurement parameters.

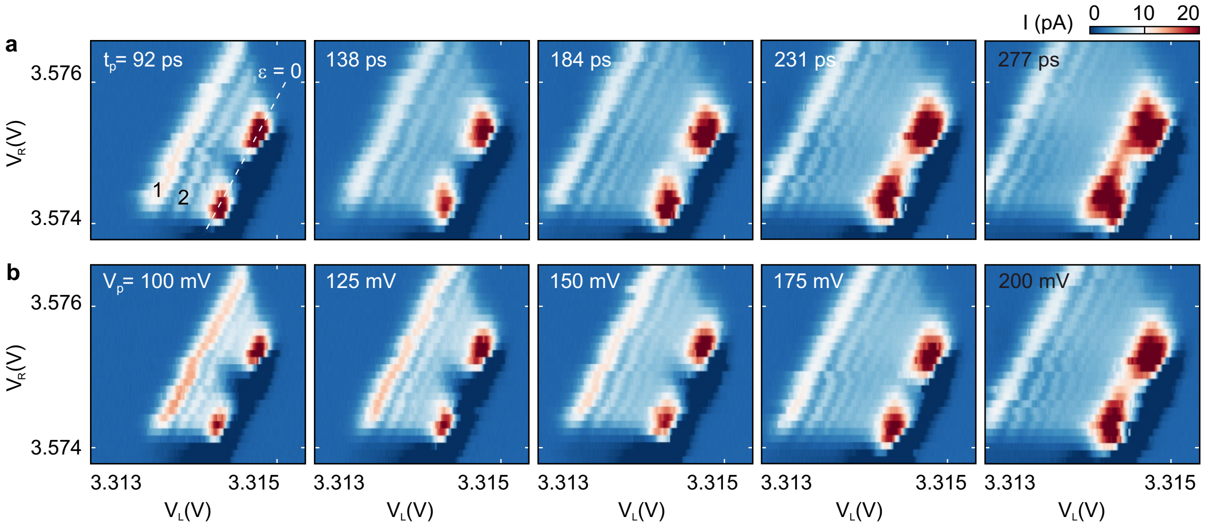

Supplementary Fig. 5. **Charge stability diagrams with applied square pulse. a** Charge stability diagrams of a triple point with a pulse of amplitude $V_\mathrm{p} = 200\,\mathrm{mV}$ as set at the arbitrary waveform generator (AWG) for different pulse durations t_p. **b** Charge stability diagrams of a triple point with a pulse of duration $t_\mathrm{p} = 231\,\mathrm{ps}$ and different pulse amplitudes V_p.

Supplementary Fig. 5 shows the evolution of interference fringes in the charge stability diagram of a triple point, as a function of (a) the pulse duration t_p and (b) the pulse amplitude V_p. As explained in the main manuscript, these two parameters change the relative phase that the two parts of a wave function, split in an LZSM experiment, acquire before interfering with each other. Supplementary Fig. 5a shows a series of triple points where t_p is increased from $92\,\mathrm{ps}$ to $277\,\mathrm{ps}$. Due to the finite rise time of the pulse of $t_\mathrm{r} \approx 140$ ps, the effective pulse amplitude A_p applied to the sample is reduced if $t_\mathrm{p} \lesssim t_\mathrm{r}$. Thus, less interference fringes can be observed in the triple points recorded at $t_\mathrm{p} = 92$ ps and $t_\mathrm{p} = 138$ ps. Caused by dephasing, the fringes become less clearly defined for increasing t_p. In Supplementary Fig. 5b, a set of measurements is shown, recorded at constant $t_\mathrm{p} = 231\,\mathrm{ps}$ while varying V_p. A growing number of interference fringes can be observed for increased pulse amplitudes.

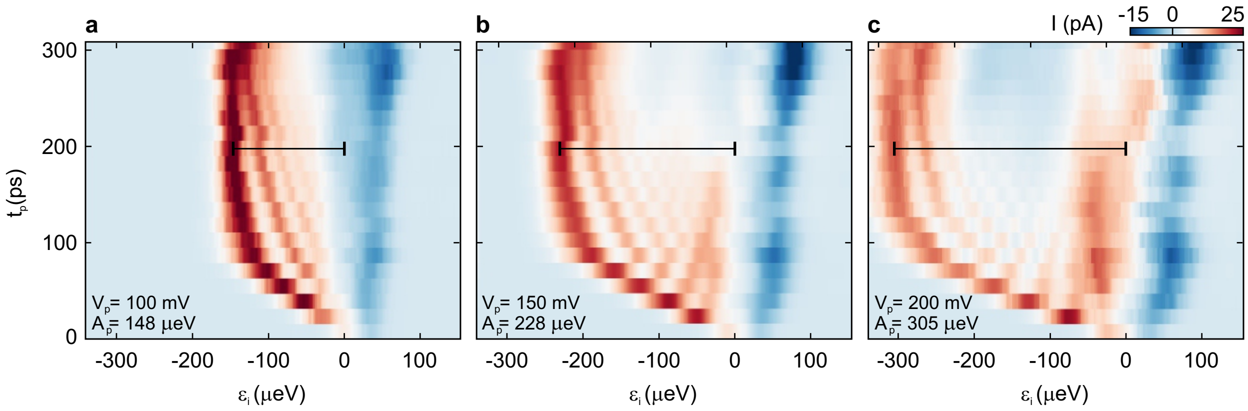

Supplementary Fig. 6. **Coherent oscillations in the time domain.** The panels show the effect of a pulse with amplitude $V_\mathrm{p} = 100\,\mathrm{mV}$ (**a**), $150\,\mathrm{mV}$ (**b**) and $200\,\mathrm{mV}$ (**c**). From the position of the first interference maximum, marked by black bars, the resulting effective amplitudes are determined to be $A_\mathrm{p} = 148\,\mu\mathrm{eV}$, $228\,\mu\mathrm{eV}$ and $305\,\mu\mathrm{eV}$, respectively. The data in panel **b** is the same as presented in Fig. 4b of the main manuscript.

The effect of changing the pulse amplitude can also be studied in the time domain. Supplementary Fig. 6 shows the results of LZSM interference experiments with the pulse duration ranging from 0 ps to 300 ps, for different values of V_p. The data sets have been acquired at the triple point shown in Fig. 4a of the main manuscript at a value of $V_\mathrm{R} = 3.368\,\mathrm{V}$. The effective pulse amplitude experienced by the sample is given by the position of the first fringe at a time $t_\mathrm{p} \gg t_\mathrm{r}$. As indicated by the black bars in Supplementary Fig. 6, one finds values of $A_\mathrm{p} = 148\,\mu\mathrm{eV}$, $228\,\mu\mathrm{eV}$ and $305\,\mu\mathrm{eV}$, respectively. This shows that A_p scales linearly with V_p, as would be expected, with a conversion factor of $\beta \approx 1.50 \pm 0.02\ \mu\mathrm{eV/mV}$.

The influence of the pulse duration, in turn, can be demonstrated in the amplitude domain. Supplementary Fig. 7 shows three amplitude-dependent measurements at different t_p. The clarity of the fringes diminishes as the pulse duration increases, indicating a progressive loss of coherence as the time between successive LZ transitions increases.

The data sets can strongly vary from triple point to triple point, as changing the charge occupation in the DQD influences both the tunnel rates to the leads [4] and the level spectrum [5, 6]. Supplementary Fig. 8a shows a data set of amplitude-dependent LZSM interference measured at the triple point shown in Supplementary Fig. 8b. Supplementary Fig. 9 depicts LZSM measurements at a different charge occupation where the overall tunneling rates have decreased. This is evident from the reduced current as well as the reduced LZSM

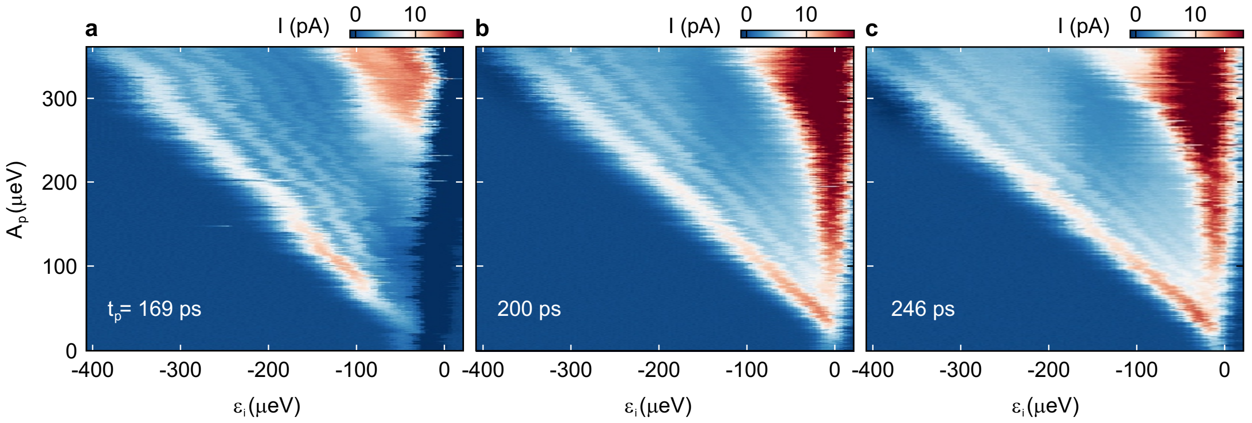

Supplementary Fig. 7. **Coherent oscillations in the amplitude domain.** The panels show the effect of a pulse with an duration of $t_\mathrm{p} = 169\,\mathrm{ps}$ (**a**), $200\,\mathrm{ps}$ (**b**) and $246\,\mathrm{ps}$ (**c**). Fig. 5a in the main manuscript shows a close-up of the data in panel **b**. The clarity of the interference fringes diminishes for increasing t_p due to dephasing.

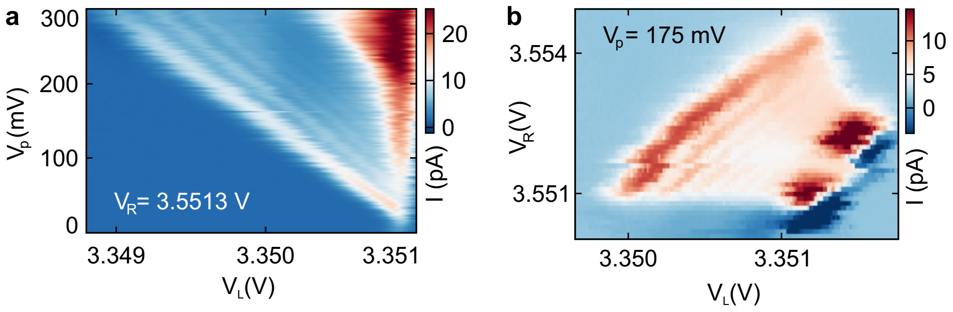

Supplementary Fig. 8. **Coherent oscillations at a different charge carrier occupation. a** Coherent oscillations in the amplitude domain complementary to the data shown in Fig. 5a of the main manuscript. A pulse with a width of $t_\mathrm{p} = 200\,\mathrm{ps}$ was applied. **b** The triple point at which the data set in **a** was recorded. Here, a pulse of $V_\mathrm{p} = 175\,\mathrm{mV}$ and $t_\mathrm{p} = 231\,\mathrm{ps}$ was applied, while $V_\mathrm{R} = 3.5513\,\mathrm{V}$.

oscillation period in detuning (a) and pulse duration t_p (b).

Supplementary Fig. 10 shows a LZSM dataset obtained from a second device fabricated with a similar device geometry.

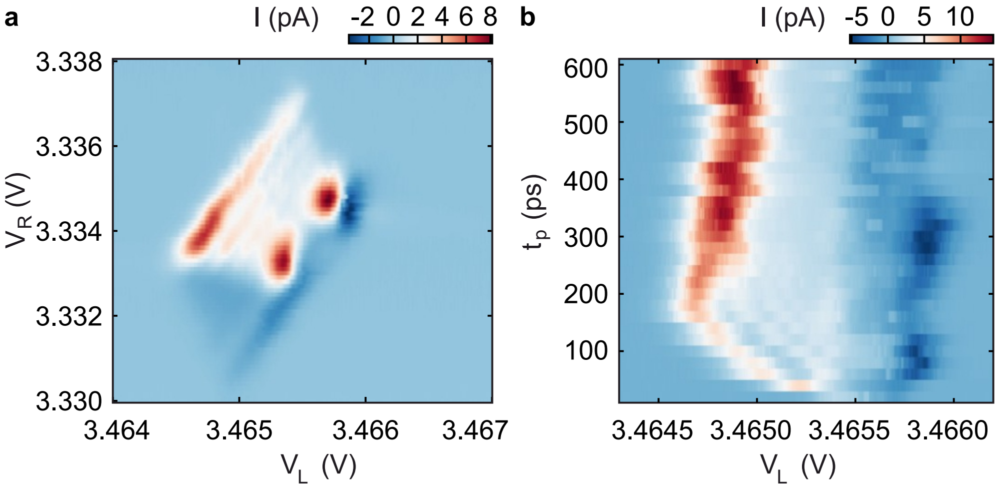

Supplementary Fig. 9. **Additional data set for lower tunnel coupling.** **a** Triple point with applied square pulse $t_\mathrm{p} = 160\,\mathrm{ps}$, $t_\mathrm{i} = 5\,\mathrm{ns}$ and $V_\mathrm{p} = 100\,\mathrm{mV}$. **b** Line cut along the x-axis in a at $V_\mathrm{R} = 3.334\,\mathrm{V}$ as function of the pulse duration t_p.

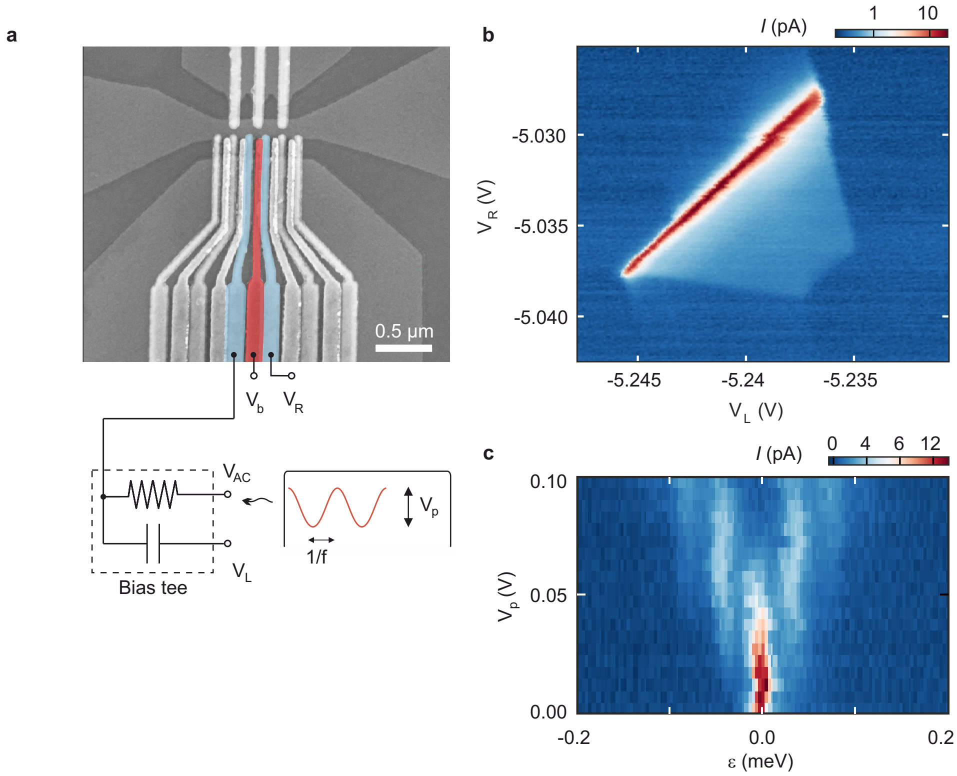

Supplementary Fig. 10. **Additional data set from a second device.** **a** False color scanning electron microscope image of the gate structure of the device. The DQD is formed below the two FGs (blue) by applying the voltages V_{L} and V_{R}. Additionally, a sine pulse can be applied to the left FG (V_{AC}). **b** Charge stability diagram showing the triple point of the charge transition $(1,0)-(0,1)$ with a bias voltage of $V_{\mathrm{SD}} = 1\,\mathrm{mV}$ and $V_{\mathrm{b}} = -7.4\,\mathrm{V}$. **c** Cut through the triple point in panel b as a function of the amplitude of the sine pulse with a frequency of $f = 10\,\mathrm{GHz}$.

IV. REMARK ON FOURIER TRANSFORM OF THE COHERENT OSCILLATIONS

Note that in Ref. [7], the decoherence time is deduced from the Fourier transform of the excitation probability P, whereas in our experiment, the measured observable is the current I. Due to the applied readout scheme, the current is proportional to the excitation probability, i.e. $I = bP$ with the proportionality factor b. This implies $I_{\mathrm{FT}} = bP_{\mathrm{FT}}$ and hence $\ln|I_{\mathrm{FT}}| = \ln|b| + \ln|P_{\mathrm{FT}}|$. This validates that $T^{*}_{2,\mathrm{FT}}$ can be determined from $\ln|I_{\mathrm{FT}}|$ according to Eq. (5) in the main manuscript.

---

[1] A. Mavalankar, T. Pei, E. M. Gauger, J. H. Warner, G. A. D. Briggs, and E. A. Laird, Photon-assisted tunneling and charge dephasing in a carbon nanotube double quantum dot, Phys. Rev. B **93**, 235428 (2016).

[2] J. R. Petta, A. C. Johnson, C. M. Marcus, M. P. Hanson, and A. C. Gossard, Manipulation of a Single Charge in a Double Quantum Dot, Phys. Rev. Lett. **93**, 186802 (2004).

[3] K. D. Petersson, J. R. Petta, H. Lu, and A. C. Gossard, Quantum Coherence in a One-Electron Semiconductor Charge Qubit, Phys. Rev. Lett. **105**, 246804 (2010).

[4] T. Ihn, *Semiconductor Nanostructures: Quantum states and electronic transport* (OUP Oxford, 2009).

[5] S. Möller, L. Banszerus, A. Knothe, C. Steiner, E. Icking, S. Trellenkamp, F. Lentz, K. Watanabe, T. Taniguchi, L. I. Glazman, V. I. Fal'ko, C. Volk, and C. Stampfer, Probing Two-Electron Multiplets in Bilayer Graphene Quantum Dots, Phys. Rev. Lett. **127**, 256802 (2021).

[6] M. Eich, F. Herman, R. Pisoni, H. Overweg, A. Kurzmann, Y. Lee, P. Rickhaus, K. Watanabe, T. Taniguchi, M. Sigrist, T. Ihn, and K. Ensslin, Spin and Valley States in Gate-Defined Bilayer Graphene Quantum Dots, Phys. Rev. X **8**, 031023 (2018).

[7] M. S. Rudner, A. V. Shytov, L. S. Levitov, D. M. Berns, W. D. Oliver, S. O. Valenzuela, and T. P. Orlando, Quantum Phase Tomography of a Strongly Driven Qubit, Phys. Rev. Lett. **101**, 190502 (2008).
